# *Fusarium musae* Infection in Animal and Plant Hosts Confirms Its Cross-Kingdom Pathogenicity

**DOI:** 10.3390/jof11020090

**Published:** 2025-01-24

**Authors:** Valeria Tava, Agustin Reséndiz-Sharpe, Eliane Vanhoffelen, Marco Saracchi, Paolo Cortesi, Katrien Lagrou, Greetje Vande Velde, Matias Pasquali

**Affiliations:** 1 Department of Food, Environmental and Nutritional Sciences, University of Milan, 20133 Milan, Italy; valeria.tava@unimi.it (V.T.); marco.saracchi@unimi.it (M.S.); paolo.cortesi@unimi.it (P.C.); 2 Department of Imaging and Pathology, KU Leuven, 3000 Leuven, Belgium; agustin.resendizsharpe@kuleuven.be (A.R.-S.); eliane.vanhoffelen@kuleuven.be (E.V.); 3 Department of Microbiology, Immunology and Transplantation, KU Leuven, 3000 Leuven, Belgium; katrien.lagrou@kuleuven.be; 4 Department of Laboratory Medicine and National Reference Center for Mycosis, UZ Leuven, 3000 Leuven, Belgium

**Keywords:** *Fusarium musae*, cross-kingdom pathogens, infection models, *Galleria mellonella*, banana fruit, infection proof

## Abstract

*Fusarium musae* is a pathogen belonging to the *Fusarium fujikuroi* species complex, isolated from both banana fruits and immunocompromised patients, therefore hypothesized to be a cross-kingdom pathogen. We aimed to characterize *F. musae* infection in plant and animal hosts to prove its cross-kingdom pathogenicity. Therefore, we developed two infection models, one in banana and one in *Galleria mellonella* larvae, as a human proxy for the investigation of cross-kingdom pathogenicity of *F. musae*, along with accurate disease indexes effective to differentiate infection degrees in animal and plant hosts. We tested a worldwide collection of *F. musae* strains isolated both from banana fruits and human patients, and we provided the first experimental proof of the ability of all strains of *F. musae* to cause significant disease in banana fruits, as well as in *G. mellonella*. Thereby, we confirmed that *F. musae* can be considered a cross-kingdom pathogen. We, thus, provide a solid basis and toolbox for the investigation of the host–pathogen interactions of *F. musae* with its hosts.

## 1. Introduction

*Fusarium musae*, a pathogenic species within the *Fusarium fujikuroi* species complex, was formally recognized in 2011 as a species closely related to but distinct from *F. verticillioides* [1]. It was first identified as a plant pathogen and recognized as one of the causative agents of crown rot in banana fruits. Cases of infection with *F. musae* have been reported in banana fruits in Hungary [2], Neotropical countries [3,4,5], and the Philippines [1]. Later, it was also described as a pathogen in human patients, where it caused keratitis and skin infections, as well as systemic infections in immunocompromised patients [6,7,8,9]. Cases of infection with *F. musae* have been reported in Italy, Greece [6], France, Belgium [9], and the USA [10]. Isolation of *F. musae* from different hosts raised suspicion that *F. musae* could be a potential cross-kingdom pathogen, further strengthened by its ability to cause infection in both bananas and humans. Plant fungal pathogens are already known as an emerging danger to both food production and ecosystem balance by causing food losses, impacting agricultural productivity, and contaminating food with harmful mycotoxins. But recently, it has become evident that some agricultural fungi may have evolved the ability to infect human hosts, posing direct infection risks and further endangering human health. It is yet unclear if and how *F. musae* transfers from one host to the other. Triest and Hendrickx suggested a role for bananas as carriers of spores leading to diseases in immunocompromised patients in banana-consuming countries [8].

So far, there is no experimental proof of the ability of *F. musae* plant strains to invade an animal (human) host and vice versa, and it has not been shown that *F. musae* possesses the essential virulence factors to cause infection in both a plant and animal host. It is, nevertheless, important to prove infection in relevant hosts across different kingdoms to confirm cross-kingdom pathogenicity. This process ensures compliance with Koch’s postulates, which are essential for confirming the pathogenicity of a species on specific hosts [11,12]. Indeed, exploring the literature about fungal cross-kingdom pathogens, the classification of a pathogen as cross-kingdom is mainly based on isolation from different hosts and taxonomic criteria that are constantly evolving and need frequent checks and updates. Experimental evidence supporting infection across multiple hosts is limited, with only a few studies offering such data [13,14]. Experimentally demonstrating the ability of a fungal species to cause infection in the host is a crucial step in the characterization and classification of the species as a pathogen.

On the other hand, by setting up relevant experimental model systems, one can investigate the virulence factors that make up a species’ ability to infect hosts belonging to different kingdoms. Moreover, applications can be envisaged for developing strategies that may prevent infection risks from contaminated food and agricultural losses.

With this work, we provide the first experimental evidence that a collection of *F. musae* strains with different host origins can infect both animals and plants. We aimed to evaluate and compare *F. musae* pathogenicity across kingdoms, demonstrating that strains isolated from different hosts have the ability to invade both kingdoms. To reach this goal, we established two infection models that could represent the plant host and the animal host [15,16,17,18,19].

## 2. Materials and Methods

### 2.1. Fungal Strains

For this work, we used a collection of nineteen strains of *Fusarium musae*, which included strains isolated from different hosts and countries of origin (Table 1).

### 2.2. Temperature Assay In Vitro

To determine the potential ability of *F. musae* strains to grow at different temperatures, we incubated all strains on Petri dishes containing V8 media (200 mL/L V8 juice Campbell, UK; 2 g/L CaCO_3_ Carlo Erba Reagents S.r.l, Cornaredo, MI, Italy; 15 g/L agar NeoFroxx GmbH, Einhausen, Germany) at 24 °C and 37 °C for five days. Growing rate was assessed by measuring the diameter of the colony with a ruler on the reverse of the plate; for each colony, we calculated the average value of two measurements at 90° angles to each other. We considered the average value of tree replicates.

### 2.3. Banana Fruit Infection

For the infection in banana fruits, we considered 20 infection sites for each strain: 4 banana fruits, each infected with 5 sterile toothpicks as previously described [20]. Infection was performed with the entire collection of *F. musae* strains. Control groups with sterile water and *Fusarium oxysporum* f.sp. *lycopersici* were included.

**Figure 1:**
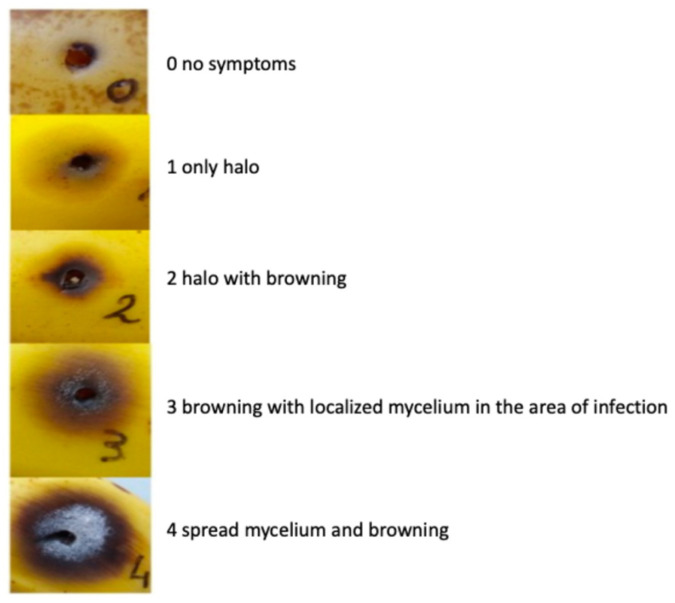
Scale of browning in banana fruits. Each spot was awarded a number from 0 to 4 based on halo, browning, and mycelium formation in order to assess the level of disease caused by each strain on bananas.

Strains were recovered from storage on V8 media plates by inoculating a few pieces of mycelium into CMC liquid media (15 g/L carboxymethylcellulose sodium (CMC) Sigma-Aldrich (St. Louis, MO, USA); 1 g/L NH_4_NO_3_ Carlo Erba Reagents S.r.l, Cornaredo, MI, Italy; 1 g/L KH_2_PO_4_ Sigma-Aldrich; 0.5 g/L MgSO_4_-7H_2_O Sigma-Aldrich; 1 g/L Yeast Extract Thermo Fisher Scientific Inc. (Waltham, MA, USA)) and incubating at 25 °C in the dark while stirring at 150 rpm. After 5 days, spores were harvested by filtering through one-fold Miracloth, and concentrations were estimated using a Burker counting chamber to obtain the concentration of 10^5^ spores/mL for infection. Fresh conidia were used for every experiment. Healthy, organic bananas of the cultivar Cavendish with similar maturation level (green banana) obtained from Ecuador were used on the infection day; they were sterilized by immersion in 0.7% sodium hypochlorite (3 min), washed in deionized sterile water (3 min), and dried under a sterile laminar flow hood. Sterile toothpicks were immersed for 2 min in conidia solution and skewered in bananas for infection. Five toothpicks were evenly positioned per fruit, deep enough to pierce the peel and reach the pulp surface. Subsequently, fruits were covered by plastic bags and incubated at 20 °C in the dark for 10 days. After 10 days, levels of infection were estimated by measuring the diameter of the spots growing on the banana fruits, as described in Figure 1.

Infections were performed in three independent experimental repeats. In two of the replicates, all five toothpicks per fruit were soaked in either a conidia suspension from the same strain or in water. In the third replicate, each of the five infection points on each fruit received a different treatment (either conidia suspension or water) to account for any effect the individual fruits might have on infection severity.

We assessed infection in banana fruits by formulating a disease severity index that best represents the disease considering the diameter of the halos as well as browning in each infection point. The index was calculated as follows:

$$disease\;severity\;index=\frac{\left[{\left(S\right)}^{2}+\frac{D\;}{cm}\right]}{2}$$

*S* = based on the scale of browning; *D* = diameter of the spot (cm).

### 2.4. G. mellonella Larvae Infection Model

For infection in *G. mellonella*, we bought specimens of *G. mellonella* at the larval final stage from Biosystems Technology Ltd. (Tanners’ Yard, Crediton, UK). Larvae were maintained at 16 °C and infected within 7 days from arrival at our laboratory. Only healthy larvae were selected and used for the infection. Fungal strains listed in Table 1 were recovered from long-term storage at −80 °C by inoculating them in Petri dishes containing solid Sabouraud agar (Sigma-Aldrich) and incubating at 24 °C for 5 days. On the fifth day, the conidia were harvested directly from the plate by gently flooding the surface with 1 mL of phosphate-buffered saline (PBS) solution (Thermo Fisher Scientific). Concentrations were estimated using a Burker chamber, and appropriate dilutions were made to obtain the required concentration of 10^5^ conidia/mL for infection. Per strain, groups of 10 larvae were infected with 1 × 10^5^ conidia/mL in a volume of 10 μL of each inoculum suspension. The infection was performed with a 1 mL insulin syringe into the last left proleg of the larva. Larvae were incubated at the desired temperature (24 °C, 28 °C, or 37 °C) in Petri dishes for up to 7 days. PBS and *F. oxysporum* were used as controls.

### 2.5. Temperature Assay in G. mellonella Larvae

Seven representative strains were chosen for temperature assay in *G. mellonella*. We chose plant strains F31, NRRL 28893, and MUCL 51371 and human strains IUM 11-0508, NRRL 43601, IHEM 20180, and NRRL 43658. The infection was performed as described before, and larvae were incubated at 24 °C, 28 °C, and 37 °C for 7 days.

Twice a day, all larvae were observed, and each larva was awarded a health score based on the level of activity, pigmentation, cocoon formation, and state of life according to the scoring system established by Loh et al. (Table 2) [21].

Infections were carried out independently two times. After scoring, dead larvae were removed from the Petri dishes and excluded for the following checkpoints. Dead larvae were placed at −20 °C for at least 48 h before being disposed of.

### 2.6. Infection Assay in G. mellonella Larvae

Infection in *G. mellonella* was performed with the entire collection of *F. musae* strains using groups of 10 larvae infected with 10 μL of conidia suspension at 1 × 10^5^ conidia/mL, as described before. Larvae were incubated at 28 °C for 7 days, and the level of infection was scored daily [21].

To be able to compare infection severity in the *G. mellonella* model to the data obtained from the banana model, we transformed the health scores used from evaluating larval health into a measure of disease severity as follows:

$$\mathrm{Disease}\;\mathrm{severity}\;\mathrm{index}=\frac{\left[\left(9-S\right)\times N\right]}{10}$$

S (sum)= A + M + C + L; A (activity) = value from 0 to 3; M (melanization) = value from 0 to 4; C (cocoon formation) = value from 0 to 1; L (survival) = 0 (dead), 1 (alive); N = number of dead larvae.

The disease severity index represents the severity of the disease on the final observation day in each infection point, where single infection points are represented by single larvae. Consequently, the disease severity index was assigned to each larva and calculated based on “S”, which corresponds to the health score obtained on the 7th day post-infection [21], while “N” corresponds to the total number of larvae killed by the strain, and with 10 in the denominator, we account for the standard inclusion of 10 larvae per experimental group.

Specifically, we consider larval death a strong marker of disease severity; thus, we gave it greater weight in the formula. The resulting value reflects the overall health status of the larvae at the end of the observation period. If no larval deaths are observed 7 days post-infection, this suggests that the group remains largely healthy and that the infectious strain exhibits low pathogenicity.

### 2.7. Statistical Analysis

Statistical analysis of infection of bananas and *G. mellonella* larvae was performed on the disease severity indexes obtained. The values obtained were normalized by dividing them by the average value obtained for strain F31 (used as reference strain) and plotted using the data analysis framework implemented in the online software Estimation Stats [22], with H_2_O or *F. oxysporum* as shared control under default parameters. Statistical analysis was also performed by the data analysis framework estimation stats.

For the health score in *G. mellonella*, GraphPad Software (Boston, MA, USA, version 8.0.2) was used to plot data obtained from infection at different temperatures, and mixed-effects two-way ANOVA analysis was performed to analyze longitudinal data. Survival analysis was also performed on *G. mellonella* infection using the log-rank (Mantel–Cox) test; the curve was plotted with the Kaplan–Meier method using SPSS Statistics (version 27).

For quantitative comparison of data from banana and larvae infection, data plots and statistical analysis were performed using Superplotsofdata [23].

## 3. Results

### 3.1. F. musae Can Grow at 24 °C as Well as at 37 °C

To support the hypothesis of *F. musae* as a potential cross-kingdom pathogen, we first tested in vitro the ability of *F. musae* strains to survive and spread at 24 °C and 37 °C. At both temperatures, all *F. musae* strains were able to grow, confirming the ability of the species to proliferate at environmental temperature as well as at the human body’s physiological temperature (Table S1). Strain F31 (banana) and strain IUM 11-0507 (human) showed equal growth rates at both temperatures with no statistically significant difference in the diameter of the colony. All the other strains grew significantly slower at 37 °C, observed as a reduction in the diameter of the colonies compared to the growth at 24 °C (Table S1). Overall, the host origin of the strains (human or banana) and their geographical origin had no evident effect on the growth of the colonies at different temperatures. In vitro results demonstrated that *F. musae*, regardless of being isolated from plant or human hosts, possessed the ability to grow at 24 as well as 37 °C, which supports the fact that *F. musae* is a potential cross-kingdom pathogen.

### 3.2. F. musae Is Pathogenic to Banana Fruits

To prove and quantify the capability of *F. musae* to cause infection in a plant pathosystem, we established an infection model with banana fruits. We chose the Cavendish banana fruit, the primary cultivar affected by *F. musae* and the main banana cultivar transported and distributed in banana-consuming countries.

**Figure 2:**
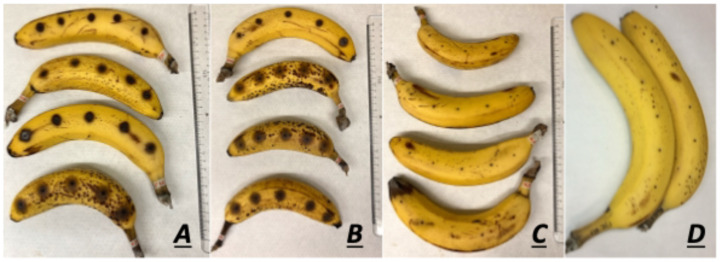
Visual representation of the symptoms observed in banana fruits. Formation of brown spots surrounding the point of toothpick insertion in bananas after 10 days of infection with 10^5^ conidia/mL of strains F31 (**A**) and IUM 11-0508 (**B**). No symptoms are visible in infection with H_2_O (**C**). No external symptoms were observed in bananas at 10 days post inoculation with *F. oxysporum* (**D**).

All nineteen strains presented brown halos surrounding the point of toothpick insertion, with some cases also showing mycelium formation (Figure 2).

**Figure 3:**
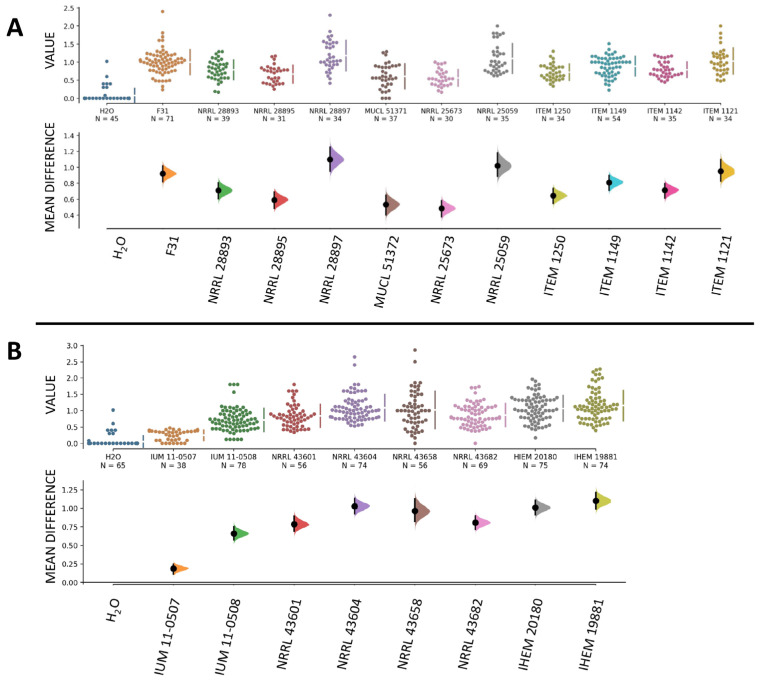
Disease severity in banana fruits caused by our collection of *F. musae* strains. Graphs were made with the data analysis framework Estimation Stats with default parameters; H_2_O was used as shared control. (**A**) Disease severity indexes of strains isolated from bananas. (**B**) Disease severity indexes of strains isolated from human patients. In both (**A**) and (**B**), the upper graphs refer to the distribution of the disease severity indexes, and the bottom graphs refer to the mean differences in value between samples inoculated with *F. musae* spores and with H_2_O, with error bars indicating a 95% confidence level. Each dot indicates a single measure.

Additionally, disease severity indexes of all tested strains were significantly higher compared to the infection with H_2_O used as control (Figure 3), demonstrating that they were all able to cause significant disease in banana fruits. Specifically, strain NRRL 19881 (human, France) caused the highest level of infection, followed by NRRL 28897 (banana, Mexico) and NRRL 43604 (human, USA), while the lowest disease was caused by strain IUM 11-0507 (human, Greece) followed by NRRL 20673 (banana, Guatemala).

**Figure 4:**
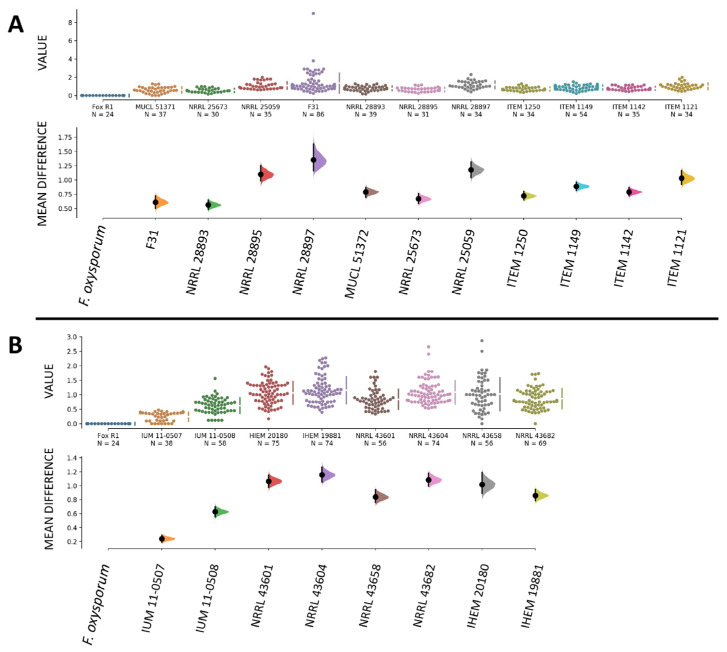
Comparison between infection in banana fruits with *F. oxysporum* and *F. musae*. Graphs were made using the data analysis framework Estimation Stats with default parameters; *Fusarium oxysporum* f.sp. *lycopersici* R1 strain was used as shared control. (**A**) Disease severity indexes of strains isolated from banana. (**B**) Disease severity indexes of strains isolated from human patients. In both (**A**) and (**B**), the upper graphs refer to the distribution of the disease severity indexes, and the bottom graphs refer to the mean difference in value between samples inoculated with *F. musae* spores and with *F. oxysporum* spores, with error bars indicating a 95% confidence level. Each dot indicates a single measure.

Overall, infection levels were comparable among all strains of our collection, and we did not observe differences in infection severity caused by strains isolated from plants and humans, as all showed similar abilities to invade a plant host such as the banana fruit. When comparing the pathogenicity of *F. musae* with the pathogenicity of *F. oxysporum*, as expected, all strains of *F. musae* showed high levels of disease severity compared to *F. oxysporum* that caused no symptoms in fruits with a disease severity score equal to 0 (Figure 4).

Taken together, we presented the first experimental evidence of *F. musae* pathogenicity in a plant host, establishing a successful model for infection. All tested *F. musae* strains isolated from human patients showed significant infection in a plant pathosystem. In addition, infection levels were comparable between plant and human strains, regardless of geographical origin, suggesting that no subgroups were present within the population based on the original source.

### 3.3. F. musae Causes Observable Disease in Larvae of G. mellonella at Environmental and Mammalian Physiological Body Temperature

To prove the ability of *F. musae* to invade an animal pathosystem, we built an infection model using *G. mellonella* larvae and assessed the ability of the species to cause infection at environmental as well as mammalian physiological body temperatures. We used the great wax moth or the honeycomb moth as ethically responsible and relevant animal models with immune systems similar to mammals, which is ideal for studying host–pathogen interactions. One of the many advantages of using *G. mellonella* as a host model is that the larvae can tolerate incubation at elevated temperatures (such as 37 °C). Furthermore, as we demonstrated, *F. musae* strains grow in vitro at both environmental and human body temperatures. Therefore, we tested different incubation temperatures to establish our *F. musae*-*G. mellonella* infection model.

**Figure 5:**
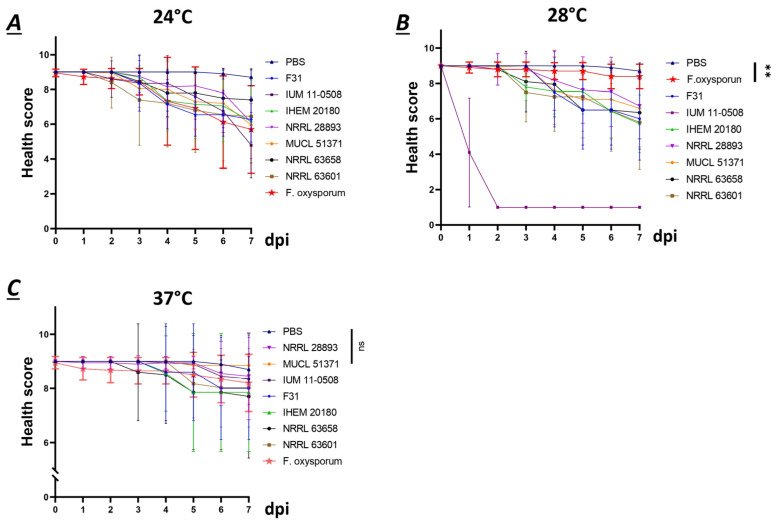
Temperature assay for *G. mellonella* infection. The virulence of seven representative strains is represented as health score at 24 °C (**A**), 28 °C (**B**), and 37 °C (**C**). Each color corresponds to a strain. Infection was initiated on day 0 and monitored for seven days. N = 10 larvae were infected per strain with 1 × 10^5^ conidia/mL, and sham infection was performed using PBS as a negative control. Mean and SD are represented in the graph. The statistics compare strains against the PBS. ns = nonsignificant; ** = *p* > 0.005.

All selected seven strains of *F. musae* were able to cause significant disease in the larvae (Figure 5).

Specifically, strains F31, NRRL 43601, NRRL 43658, and IHEM 20180 showed similar infection levels across the three temperatures. At 24 °C and 28 °C, we observed the first decline in health scores around three days post-infection, decreasing to approximately 7, which reflected reduced larval activity and the appearance of melanized spots. Health continued to decline gradually, reaching values between 5 and 6 by the end of the observation period. At 37 °C, larvae began showing symptoms 4 days post-infection, with health scores decreasing to around 7 only by the end of the observation period. Human strain IUM 11-0508 presented significantly different activity at the three temperatures, with the highest virulence at 28 °C; at this temperature, larvae were completely melanized and dead the day after infection. Plant strains MUCL 51371 and NRRL 28893 showed a significantly reduced activity at 37 °C, with no visible symptoms, compared to 24 and 28 °C, where it exhibited great disease, and health score reached values between 5 and 6. Overall, at 37 °C, the virulence of all strains was reduced compared to lower temperatures, but almost all strains presented visible disease at all temperatures, suggesting that they potentially had virulence factors needed for the infection of an animal host at environmental temperature as well as at human body temperature. When compared to infection with *F. oxyxsporum*, *F. musae* presented significantly higher virulence at 28 °C, which we identified as the optimal temperature for infection of *G. mellonella* with *F. musae*. Meanwhile, at 37 °C and 24 °C, the behavior between the two species was comparable.

Altogether, the results provided the first experimental proof of *F. musae* causing infection in an animal pathosystem at different temperatures. All strains responded similarly to the tested temperatures; strains with both plant and human origins caused evident symptoms in *G. mellonella*, making it a relevant model for investigating *F. musae*, which can be extended to other *Fusarium* species as well.

### 3.4. Human and Plant Strains of F. musae Cause Comparable Levels of Infection in G. mellonella Larvae

To observe if and how the origin of the strains could be related to a specific disease severity or phenotype in *G. mellonella*, we evaluated the disease severity caused by the entire population of *F. musae* strains.

**Figure 6:**
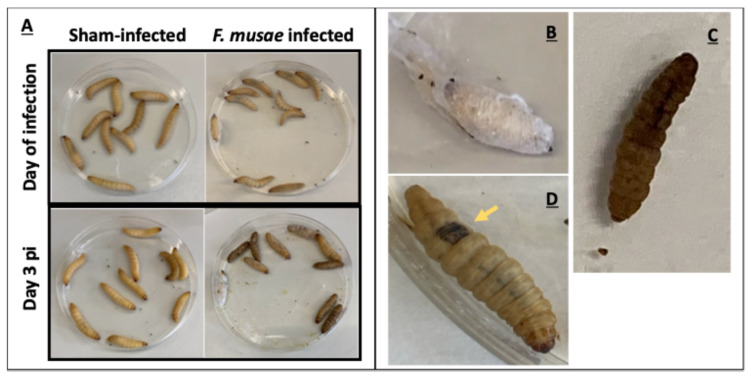
Visual examples of symptoms appearing in larvae of *G. mellonella* following infection with *F. musae* strains. Larvae were infected with 1 × 10^3^ conidia/larva in a volume of 10 μL of strain F31; injection with PBS was used as negative control. Representative pictures on day of infection and three days after infection are shown here (**A**). Symptoms observed during disease progression assessment: cocoon formation (**B**); complete melanization and consequent death (**C**); and appearance of a single dark spot (**D**).

We observed that all *F. musae* strains were able to cause symptoms; three days after the infection, a few melanized spots were already visible on numerous larvae, while larvae injected with PBS remained healthy (Figure 6). Results were consistent and reinforced by data obtained at 28 °C from the temperature assay, where only seven representative strains were included.

**Figure 7:**
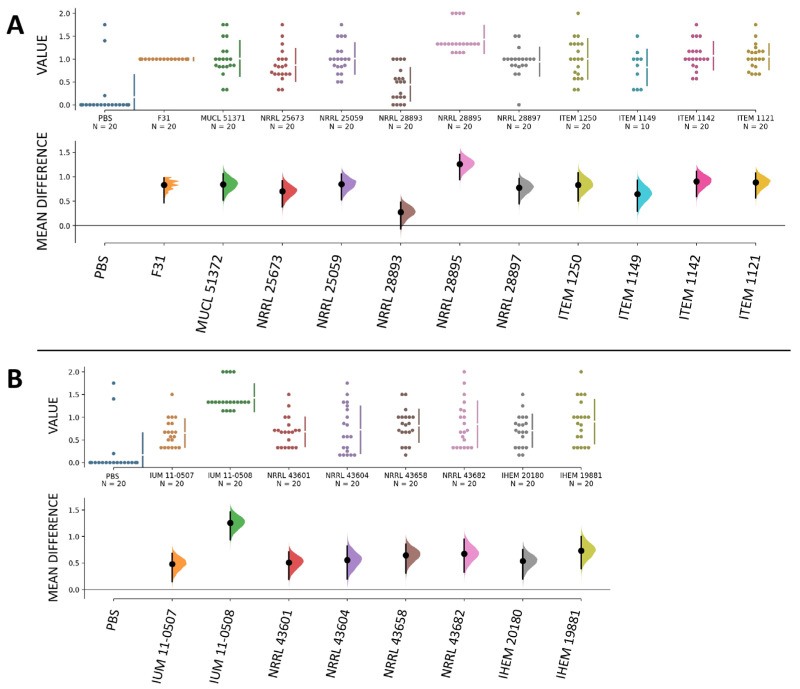
Disease severity in *G. mellonella* caused by our collection of *F. musae* strains calculated for each larva. Graphs were made with the data analysis framework estimation stats with default parameters; PBS is used as shared control. (**A**) Disease severity indexes of strains isolated from banana. (**B**) Disease severity indexes of strains isolated from human patients. In both (**A**) and (**B**), the upper graphs refer to the distribution of the disease severity indexes, and the bottom graphs refer to the mean difference in value between samples inoculated with *F. musae* spores and with PBS, with error bars indicating a 95% confidence level. Each dot indicates a single measure.

Of the nineteen *F. musae* strains tested, five (three human strains, IUM 11-0507, NRRL 43601, and NRRL 43604, and two plant strains, NRRL 28893 and ITEM 1149) showed the level of disease not statistically different from PBS (Figure 7). Strain IUM 11-0508 and NRRL 28895, isolated from human patients and banana fruits respectively, were the most virulent.

At the end of the observation period, in every group infected with *F. musae* strains, we documented the development of significant symptoms that led to larval death across all the groups.

**Figure 8:**
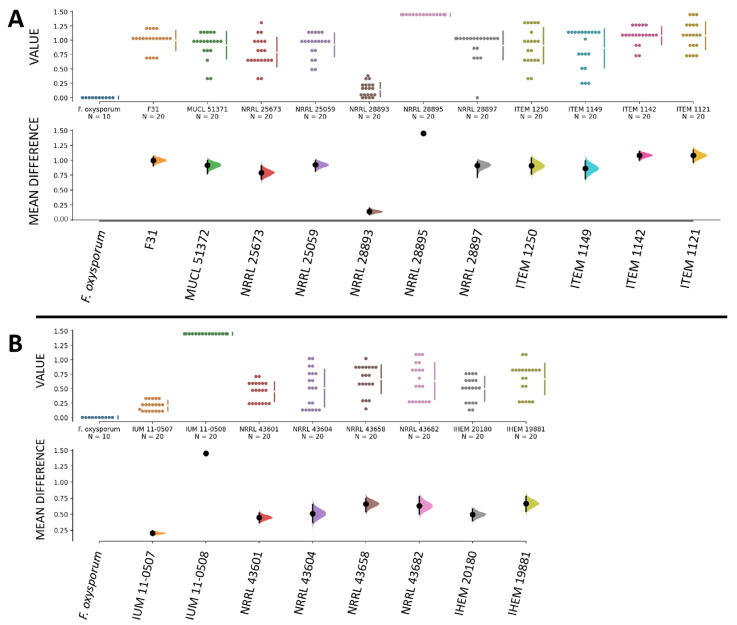
Infection in *G. mellonella* larvae with *F. musae* strains and *F. oxysporum*. The graph was made with the data analysis framework estimation stats with default parameters; *Fusarium oxysporum* f.sp. *lycopersici* R1 strain was used as shared control. (**A**) Disease severity indexes of strains isolated from banana. (**B**) Disease severity indexes of strains isolated from human patients. In both (**A**) and (**B**), the upper graphs refer to the distribution of the disease severity indexes, and the bottom graphs refer to the mean difference in value between samples inoculated with *F. musae* spores and with *F. oxysporum* spores, with error bars indicating a 95% confidence level. Each dot indicates a single measure.

At these conditions, all strains of *F. musae* showed greater levels of disease compared to the *Fusarium oxysporum* strain, which presented a disease severity index comparable to sham-infected larvae (Figure 8).

Larvae infected with the *F. oxysporum* strain displayed decreased activity in a few larvae, the appearance of a single melanized spot in two larvae in one of the groups, cocoon formation in half of the larvae, and no larvae died within 7 days post-infection. When calculating the disease severity index, infection with *F. oxysporum* resulted in an index equal to zero, comparable to sham-infected larvae. In this case, using larval death as a strong marker of disease severity obscured the representation of other symptoms. However, overall, the group remained largely healthy, and the infectious strain exhibited low pathogenicity.

Similar to what we found in banana infection, strains with different geographical origins and host isolation sources caused comparable levels of infection in *G. mellonella*, suggesting that they all had virulence factors needed for the infection of an animal host.

### 3.5. Survival Readouts of G. mellonella Larvae Infected with Human and Plant Strains of F. musae

**Figure 9:**
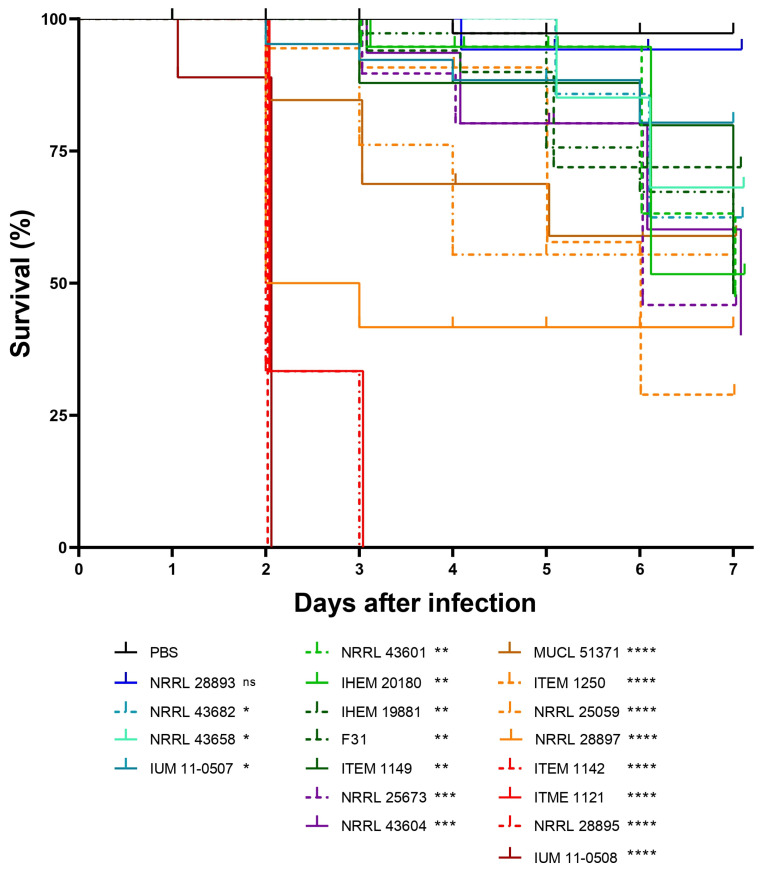
Survival plot of *F. musae* infection in *G. mellonella* larvae. Each color corresponds to a strain. Infection was initiated on day 0 and monitored for 7 days; 10 larvae were infected per strain, and average values for each timepoint are represented. Injection with PBS is used as negative control. Mean and SD are represented in the graph. The statistics compare all strains against the PBS. ns = nonsignificant; * = *p* > 0.05; ** = *p* > 0.005; *** = *p* > 0.0005; **** = *p* < 0.0001.

Given that survival analysis is still one of the most used readouts in *G. mellonella* studies and to allow for comparison with the body of the literature, we also decided to include a survival analysis in the evaluation of our infection model. Survival analysis was performed on survival data observed during the *G. mellonella* infection assay. *F. musae* infection resulted in significant mortality among the larvae (Figure 9). By 96 h post-infection, we observed a noticeable increase in the number of dead larvae, which continued to rise over the following days, indicating that both plant and human strains were capable of killing the larvae.

The virulence of the plant strain NRRL 28893 was not statistically different from the negative control. Infection with all other strains was significantly more virulent than sham-infected larvae, with no significant differences observed between strains from different origins. Overall, survival analysis confirmed that infection with *F. musae* led to a high number of dead larvae, supporting larval death as a strong marker of disease severity.

### 3.6. Quantitative Comparison of Data from Bananas and Larvae Infection

**Figure 10:**
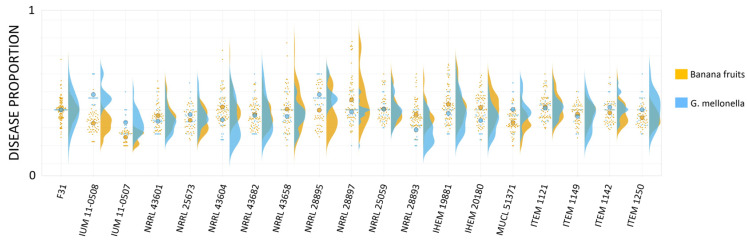
Superplot of virulence of our strains in two hosts. Overall indexes are represented in yellow when related to banana infection and in blue when related to *G. mellonella*. Small dots represent single infection points on bananas or single larvae; bigger dots represent mean values. Infection with strain F31 is considered as a reference. Data obtained from the infection with the entire collection of strains are normalized by dividing for the mean value obtained from infection scoring of the strain F31.

To study if human and plant strains showed the same behavior in both hosts and if specific trends were observable based on the isolation origin of the strains, we plotted together the data obtained from the infection of banana fruits and *G. mellonella* larvae with the entire collection of nineteen strains of *F. musae*. The distribution of data showed that infection severity in banana fruits was mostly comparable with that observed in *G. mellonella* (Figure 10).

Only human strain IUM 11-0508 showed different behavior in the two different hosts, as it was highly virulent in *G. mellonella* but less virulent in banana fruits. We did not find any specific interdependence between hosts or geographical origin of the strains and their virulence.

## 4. Discussion

The field of fungal cross-kingdom pathogens is still in its infancy. A conspicuous number of species has been identified as pathogenic to organisms from more than one kingdom [13,24]. In most cases, classification as a cross-kingdom pathogen is performed based on the isolation from different hosts of strains with shared taxonomic criteria. To qualify as a cross-kingdom pathogen, evidence of infection in the different hosts is essential. Studies verifying Koch’s postulates in diverse hosts are limited to pathogens that are considered cross-kingdom

*F. musae* is a putative cross-kingdom pathogen; in fact, strains isolated from humans and bananas are part of common taxa [1,25,26,27]. However, the pathogenicity of *F. musae* has not yet been experimentally verified, as is required by Koch’s postulates. Therefore, in this work, we aimed to prove experimentally if *F. musae* could be confirmed as a cross-kingdom pathogen by validating infection models for the investigation of its pathogenicity in both plant and animal kingdoms.

Our results showed that all *F. musae* strains of our collection, independently of their isolation source, could grow in vitro at 25 °C as well as at higher temperatures such as 37 °C, suggesting that they are all well adapted to occupy different ecological niches and share virulence factors needed to spread at environmental temperature but also at a human body temperature.

Indeed, pathogenicity in different host models’ environments is an important step to understanding *F. musae* as a cross-kingdom pathogen; therefore, our next step was to identify the most suitable hosts for this investigation. Banana fruits are the only known hosts of *F. musae* in the plant kingdom, and the fungus is one of the causative agents of crown rot, which is considered one of the most important post-harvest diseases of bananas [3]. Therefore, we established an infection model for the investigation of *F. musae* using banana fruits similar to the model used for the investigation of *F. verticillioides* [20].

For the animal kingdom, we established an infection model with larvae of *Galleria mellonella*. *G. mellonella* larvae are considered a successful and ethically responsible, relevant host model for the study of host–pathogen interaction. When compared to rodent models of infection, *G. mellonella* larvae offer considerable advantages: they are easily and inexpensively obtained in large numbers, are simple to use, and are easy to maintain without special laboratory equipment. Additional major benefits are their ability to thrive at 37 °C, their ease of maintenance and manipulation, few ethical constraints, and their size, which simplify the infection [28,29,30,31,32]. The structural and functional similarities between the innate immune system of mammals and the insect immune response present an additional reason to use it for the study of human fungal pathogens [33,34,35,36,37]. Indeed, among the host model alternatives to mice, *Galleria mellonella* larvae present several favorable attributes that make it an optimal model host for the investigation of various fungi, including *Fusarium* spp., as the well-known cross-kingdom pathogens *F. solani* and *F. oxysporum* [18,29,38,39,40,41,42,43,44,45,46].

We hypothesized that temperature would play a crucial role during the infection of *G. mellonella* larvae. Consequently, we tested the ability of our collection of strains to cause infection at environmental as well as mammal physiological body temperatures. We observed that all our strains showed 28 °C as the optimum temperature for the infection, while at 37 °C, the growth of our strains was overall reduced. This is consistent with the environments from where they were isolated: bananas usually grow at temperatures ranging from 25 °C to 28 °C, while the human body temperature is not uniform throughout. While core body temperature is 37 °C, extremities and corneal tissues have lower temperatures and are more exposed to injuries, making them more susceptible to fungal infections, including those by *F. musae* [24,47].

We did not only consider the best experimental model for studying *F. musae* pathogenicity but also the methodology to evaluate the extent of disease caused in host model systems representing plant and animal kingdoms. To evaluate banana fruit infection, we established a semiquantitative scoring system based on halo, browning, and mycelium formation. For evaluating disease severity in *G. mellonella*, survival analysis is the most used readout for the study of virulence of fungi of clinical interest, but it gives a binary result that does not allow us to observe other or milder symptoms of infection. A more sophisticated measure exists that accounts for not only life or death but several aspects of larval health [21]. Melanization is part of *G. mellonella*’s humoral system; it is a complex enzymatic cascade where melanin is synthesized due to wound and pathogen encapsulation, and usually, it correlates with the death of the wax worm soon after. The degree of melanization can depend on the infecting pathogen [41]. Some pathogens cause profound and uniform melanization, whilst others cause more subtle color changes, which can be difficult to interpret. Higher activity corresponds to a healthier wax worm, while cocoon formation and activity of the larvae indicate good health of the individual [17,21].

To better represent disease severity in our infection models and enable comparison of the virulence level of single strains, we established disease severity indexes. The banana disease severity index takes into account the diameter and the level of browning of the spots growing on the fruits, while the *G. mellonella* severity index considers the importance of larval death as well as other symptoms that can be linked to specific mechanisms involved in pathogen–host interaction. These indexes allowed us to assess and compare the virulence of individual strains in both bananas and *G. mellonella* models, observing possible differences in the interaction with the two different hosts.

Based on this novel experimental model with the disease severity quantification framework we established, we verified that strains of *F. musae* with different hosts and geographical origins could cause a high level of disease, and most of the strains did not discriminate between the host tested, as they were able to cause comparable levels of disease in both banana fruits and *G. mellonella* larvae. Interestingly, the human strain IUM 11-0508 showed different infections of the two hosts, causing high disease levels in *G. mellonella* while being a weak pathogen of bananas. It is known that different model organisms may also differentiate infection characteristics within a species (as shown in *Aspergillus fumigatus* [48]). We are, therefore, aware that the animal model selected here, despite its advantages and extensive use [48,49], does not fully mimic the host response of a mammalian host; therefore, future studies addressing the behavior of the fungus in mammalian models are warranted.

Overall, all the tested strains of *F. musae* consistently showed high infection in both our host models, with infection levels that were comparable among plant- and human-derived strains. Additionally, variations in symptoms and degrees of infection were observed among *F. musae* strains in both hosts. Together, these findings indicate that *F. musae* virulence is driven by strain specificity rather than host specificity. Our phenotypic tests allowed us to identify strains that have peculiar behavior against the different hosts, such as IUM 11-0508, as well as strains that grow with difficulty at temperatures required for human infection, such as NRRL 28897 and ITEM 1149. A genomic approach trying to link these characteristics to the genetic background of the strains is warranted as this will guide our comprehension of virulence factors underlying pathogenicity across kingdoms.

Considering the similarities in defense systems of plants and humans [47,48,49,50,51,52], it is crucial to further explore the strategies evolved by *F. musae* that allow it to invade hosts belonging to different kingdoms and the underlying genetic determinants of infection. Indeed, *Fusarium* species are an interesting model to explore from the One Health perspective, given the complexity of these pathogen–host interactions [53].

## 5. Conclusions

We established here two relevant infection models, one involving banana fruits and the other one involving *G. mellonella* larvae, successfully demonstrating the cross-kingdom pathogenicity of *F. musae* and providing a solid procedure to demonstrate the virulence of *F. musae* strains in different infection models.

## Figures and Tables

**Table 1:** *F. musae* collection. For each strain, the country, the host from which it was isolated, and the reference to first description are indicated. ^a^ Five strains isolated from bananas and ^b^ four strains isolated from human patients were obtained from the ARS Culture Collection database. ^c^ Two human strains and ^d^ one banana strain were obtained from the Belgian coordinated collections of Microorganisms collection. ^e^ Four banana strains were obtained from the Institute of Food Production Sciences in Bari, Italy. ^f^ Three strains belonged to the University of Milan.

Strain	Country	Host (Tissue)	Reference
F31 ^f^ (DSMZ 112727)	Dominican Republic	Banana (fruit)	[3]
IUM 11-0507 ^f^	Greece	Human (blood)	[6]
IUM 11-0508 ^f^	Greece	Human (cornea)	[6]
NRRL 28893 ^a^	Mexico	Banana (fruit)	[1]
NRRL 28895 ^a^	Mexico	Banana	[4]
NRRL 28897 ^a^	Mexico	Banana	[4]
NRRL 43601 ^b^	Maryland, USA	Human (skin)	[10]
NRRL 43604 ^b^	Ohio, USA	Human (nasal sinus)	[10]
NRRL 43658 ^b^	Minnesota, USA	Human (contact lens)	[10]
NRRL 43682 ^b^	Minnesota, USA	Human (cornea)	[10]
NRRL 25673 ^a^	Guatemala	Banana (fruit)	[1]
(MUCL 53204)			
NRRL 25059 ^a^	Honduras	Banana (fruit)	[1]
(CBS 624.87, MUCL 52574)			
IHEM 20180 ^c^	Brussels, Belgium	Human (sinus biopsy)	[5]
IHEM 19881 ^c^	Brest, France	Human (shoulder biopsy)	[9]
ITEM 1121 ^e^	Panama	Banana (fruit)	[9]
(MUCL 52573)			
ITEM 1142 ^e^	Ecuador	Banana (fruit)	[1]
(MUCL 53196)			
ITEM 1149 ^e^	Panama	Banana (fruit)	[1]
(MUCL 52201)			
ITEM 1250 ^e^	Canary Islands	Banana (fruit)	[1]
(MUCL 53203)			
MUCL 51371 ^d^	Philippines	Banana (fruit)	[1]

**Table 2:** Health scoring system for *G. mellonella.* Representation of the category and scores used to assess the level of disease caused by each strain in *G. mellonella* [21]. The disease scoring of each larva at every timepoint is the sum of scores obtained for each category.

Category	Description	Score
Activity	No activity	0
	Minimal activity on stimulation	1
	Active when stimulated	2
	Active without stimulation	3
Cocoon formation	No cocoon	0
	Partial cocoon	0.5
	Full cocoon	1
Melanization	Complete melanization (black)	0
	Dark spots on brown wax worm	1
	>3 spots on beige wax worm	2
	<3 spots on beige wax worm	3
	No melanization	4
Survival	Dead	0
	Alive	1
	**TOTAL**	**SUM**

## Data Availability

Raw data are available upon request to the corresponding author.

## Supplementary Materials

The following supporting information can be downloaded at https://www.mdpi.com/article/10.3390/jof11020090/s1, Table S1: In vitro temperature assay of *F. musae*. The table represents average values of diameters expressed in cm of the colonies measured five days after incubation at 25 °C and 37 °C in solid V8 media. Three replicates were performed for each strain at each condition. * Two replicates were performed for these strains.
