# Supplementary material for: Fusarium musae Infection in Animal and Plant Hosts Confirms Its Cross-Kingdom Pathogenicity
Source: J Fungi (Basel). 2025 Jan 24;11(2):90. doi: 10.3390/jof11020090 (PMC11856682; doi:10.3390/jof11020090)
Supplement: Supplementary file 1 [file jof-11-00090-s001.zip › jof-3359744-supplementary.pdf]

STRAIN	HOST (TISSUE)	24°C	37°C
F31	Banana (fruit)	3,65 ± 0,03	3,4 ± 0,2
IUM 11-0507	Human (blood)	2,6 ± 0,1	2,9 ± 0,14
IUM 11-0508	Human (cornea)	3,4 ± 0,1	2,8 ± 0,5
NRRL 28893	Banana (fruit)	4,52 ± 0,1	1 ± 0,05
NRRL 28895	Banana	4,25 ± 0,7	1,25 ± 0,2
NRRL 28897	Banana	4,57 ± 0,2	0,93 ± 0,02
NRRL 43601	Human (skin)	5,05 ± 0,13	1,7 ± 0,2
NRRL 43604	Human (nasal sinus)	4,6 ± 0,5	1,32 ± 0,7
NRRL 43658	Human (contact lens)	4,85 ± 0,3	1,05 ± 0,4
NRRL 43682	Human (cornea)	4,95 ± 0,3	1,38 ± 0,06
NRRL 25673 (MUCL 53204)	Banana (fruit)	4,6 ± 0,3	1,97 ± 0,6
NRRL 25059 (CBS 624.87, MUCL 52574)	Banana (fruit)	3,86 ± 0,4	1,73 ± 0,2
IHEM 20180	Human (sinus biopsy)	3,97 ± 0,06	1,65 ± 0,3
IHEM 19881	Human (shoulder biopsy)	4,93 ± 0,5	2,12 ± 1,2
ITEM 1121 (MUCL 52573)	Banana (fruit)	4,5 ± 0,7	1,57 ± 0,3
ITEM 1142 (MUCL 53196)	Banana (fruit)	4,6*	1,18 ± 0,2
ITEM 1149 (MUCL 52201)	Banana (fruit)	4,8*	0,87 ± 0,1
ITEM 1250 (MUCL 53203)	Banana (fruit)	4,98 ± 0,6	1,45 ± 0,2
MUCL 51371	Banana (fruit)	3,33 ± 1,3	1,65 ± 1,4
